# The BENEFIT Trial: Where Do We Go from Here?

**DOI:** 10.1371/journal.pntd.0004343

**Published:** 2016-02-25

**Authors:** Bernard Pecoul, Carolina Batista, Eric Stobbaerts, Isabella Ribeiro, Rafael Vilasanjuan, Joaquim Gascon, Maria Jesus Pinazo, Silvia Moriana, Silvia Gold, Ana Pereiro, Miriam Navarro, Faustino Torrico, Maria Elena Bottazzi, Peter J. Hotez

**Affiliations:** 1 Drugs for Neglected Diseases Initiative (DNDi), Geneva, Switzerland and Rio de Janeiro, Brazil; 2 The Global Chagas Disease Coalition, Barcelona, Spain; 3 IS Global and the Barcelona Centre for International Health Research (CRESIB), Barcelona, Spain; 4 Fundacion Mundo Sano, Buenos Aires, Argentina, and Madrid, Spain; 5 Fundacion Ceades and Universidad Mayor de San Simon (UMSS), Cochabamba, Bolivia; 6 Sabin Vaccine Institute and Texas Children’s Hospital Center for Vaccine Development, National School of Tropical Medicine at Baylor College of Medicine, Houston, Texas, United States of America; Yale School of Public Health, UNITED STATES

In the next five years, we can now project that 200,000 people living with Chagas disease will die from heart disease and related complications. We urgently need to redouble our efforts to identify and treat young people who are still in the early stages of their illness, but ultimately we need to find better treatments and new cures.

According to recent estimates, there are 5.7–9.4 million people living with Chagas disease (American trypanosomiasis caused by *Trypanosoma cruzi*), a neglected tropical disease and leading cause of heart disease and cardiomyopathy, especially in Latin America and the United States [1,2]. Today, less than 1% of people infected with *T*. *cruzi* have access to diagnosis and treatment [3], a consequence of the fact that Chagas disease mostly affects those living in extreme poverty and in marginal surroundings. This finding is especially sad given new information by the World Health Organization (WHO) stating that more than one-half of Chagas disease sufferers live in Latin America’s three wealthiest countries—Argentina, Brazil, and Mexico [1]. Moreover, there are hundreds of thousands of infected people living in the US, with emerging evidence for significant *T*. *cruzi* transmission in Texas [4,5]. In this sense, Chagas disease represents one of the Western Hemisphere’s greatest health disparities. Moreover, in recent years we also have seen the globalization of Chagas disease to Spain and elsewhere in Europe and worldwide [6].

For years, the community of scientists, physicians, and other health care providers and Chagas disease patients has been awaiting the results of the Benznidazole Evaluation for Interrupting Trypanosomiasis (BENEFIT) trial, which was designed to evaluate the safety and efficacy of benznidazole in patients with Chagasic cardiomyopathy [7]. Approximately 20%–30% of *T*. *cruzi*-infected individuals progress to Chagasic cardiomyopathy, a debilitating heart condition associated with conduction disturbances, heart failure, and sudden death. While it is established that benznidazole is effective in curing Chagas disease patients during their early acute phase [8–11], very few individuals are diagnosed at this stage of the disease. The BENEFIT trial aimed to determine if the 1.17 million people now living with Chagasic cardiomyopathy (WHO estimate [1]) might also experience improved clinical outcomes or even cures with benzimidazole treatment.

Unfortunately, the answer appears to be “no.” Compared to a placebo control, benznidazole did not result in a statistically significant improvement in cardiac clinical outcomes. Although the study was not sufficiently powered to show incremental benefits in cardiac outcome (on the order of 5%–15%), it is clear that our current strategies for antiparasitic chemotherapy need to be revisited for patients with evidence of Chagasic heart disease.

But there is additional bad news—the BENEFIT trial found that in both treated and placebo arms (comprising almost three thousand patients), 17%–18% died over a five-year time frame, most from cardiac complications [7]. If we extrapolate from the WHO estimate, this means that roughly 200,000 people will die from Chagasic cardiomyopathy over the next five years. To put this number in perspective, it is almost identical to the number of women living in the US who will die from breast cancer over the same period [12].

Whereas breast cancer is now linked with a highly successfully and accomplished advocacy and awareness campaign that promotes early detection and treatment, as well as research and development (R&D) into an exciting portfolio of new and innovative therapies, we are now facing almost the opposite situation with Chagas disease and cardiomyopathy. Today, there are few advocates for the millions of Chagas disease sufferers mostly living in poor and marginalized conditions. As a result, the vast majority have no access to diagnosis and treatment, and far too little is invested into R&D for new drugs, vaccines, and other tools (including tests for cure).

From our perspective, the BENEFIT trial is a wake-up call to aggressively pursue a global initiative of diagnosis, treatment, and research, emphasizing the following specific points:

## Diagnosis, Treatment, and Public Health Interventions

- Although BENEFIT highlighted the failure of benznidazole in treating Chagasic cardiomyopathy sufferers, the medicine can still work extremely well in curing patients during the early stages of the disease, especially newly infected children and young adults. In addition, recent studies have demonstrated the impact of treatment of women of child-bearing age in preventing the vertical transmission of Chagas disease [13]. Therefore, in areas of active and intense Chagas disease transmission, such as in Mesoamerica, several tropical Latin American countries (e.g., Bolivia, Colombia, Ecuador, and Venezuela), and possibly even in Texas in the US, we recommend the implementation of programs of active surveillance in order to diagnose thousands (if not millions) of young people and women of child-bearing age who could immediately benefit from benznidazole chemotherapy as a means to prevent vertical transmission and long-term cardiac complications. Young people in nonendemic countries and regions also require access to diagnosis and treatment.
- Given the overall lack of awareness (and in some cases, resources) by the governments of these nations, we recognize the heavy lifting required and uphill climb to implement and scale up diagnosis and treatment programs; however, we also believe such activities are both feasible and practical and ultimately cost-effective. In parallel, we recommend expanding the production and availability of high-quality benznidazole and nifurtimox, as well as diagnostic kits, in order to ensure these products will reach those affected and in need.
- For children and adults who have been chronically infected with *T*. *cruzi* but have not yet shown evidence of cardiac disease (so-called indeterminate stage of the disease), we need a large and well-planned study to confirm the benefits of benznidazole in preventing long-term cardiac complications.
- For adults living with advanced Chagasic cardiomyopathy, they too require diagnosis and access to essential heart medicines for improving cardiac performance and quality of life.

## Research Design

- The BENEFIT trial highlighted serious and important weaknesses in our ability to assess new drugs and therapeutics for Chagas disease and raised a number of interesting questions in regards to future clinical trial designs for new products.
- Although two-thirds of the patients in the benznidazole treatment group experienced polymerase chain reaction (PCR) conversion, meaning that their parasite loads became undetectable, their cardiac disease failed to improve. A key question from these findings is whether parasitological cure results in a different outcome in patients with indeterminate disease. We need further data analyses and studies to understand the role of parasitological cure versus other factors.
- Along similar lines, the PCR conversion rates reported for the placebo arm in the BENEFIT trial were higher than reported in recent studies [7]. Although PCR methods for the detection *of T*. *cruzi* DNA have been internationally validated [14], we may need to determine whether the trypanocidal effects of benznidazole were overestimated in the BENEFIT trial.
- The study found that clinical progression and PCR conversion was significantly higher among Chagas disease patients from Brazil compared to Colombia and El Salvador. What is the role of parasite strain or genotype differences when considering treatment options? Conversely, what about host factors, such as pharmacokinetic and bioavailability factors, or human genomic variation might account for these findings?
- The study evaluated a fixed dose of 300 mg per day and a variable duration of therapy (between 40 and 80 days) on the basis of the patient’s weight, in order to keep constant the total dose of the drug administered. However, given that there is limited information on the pharmacokinetics and pharmacodynamics of benznidazole in patients with Chagas disease, we may need to reconsider the dosing of this drug. Optimal dosing schedule for benznidazole needs to be evaluated, and we should consider further dose-finding trials.
- Our ability to assess the impact of Chagas disease medicines on improving heart disease remains severely hamstrung by the long periods of follow up and the quality and reliability of currently available biological markers indicating therapeutic responses. We do not have strong, qualified surrogate markers that can substitute for clinical outcomes. Such deficiencies represent an urgent and grand challenge of Chagas disease that demand concerted action and exploration.
- We still do not understand the role of comorbidities, including malnutrition, coinfections, and noncommunicable diseases, when treating Chagas disease patients.

## New Therapies

- Through the activities of nonprofit product development partnership, a pipeline of promising new therapies for Chagas disease has been established. They include new pediatric formulations of benznidazole, new drugs, and new benznidazole treatment regimens (including combination therapies) [15], as well as a new therapeutic vaccine linked to benznidazole chemotherapy [16]. The BENEFIT trial results, while disappointing, also present new opportunities to evaluate this next generation of new products.

The negative results of the BENEFIT trial are a potential setback for the more than 1 million people now living with Chagasic cardiomyopathy, and at least one in six will die from their disease in the next five years. In parallel, millions more are living with Chagas disease in the acute and indeterminate stages of their disease, but most of them are unaware of their condition while being denied access to diagnosis and treatment. The BENEFIT trial is a clarion call for the international community to now aggressively step up efforts to increase diagnosis, treatment, and R&D for Chagas disease.

## References

[1] Chagas disease in Latin America: an epidemiological update based on 2010 estimates. Wkly Epidemiol Rec 2015; 90: 33–43. 25671846

[2] Global Burden of Disease Study 2013 Collaborators. Global, regional, and national incidence, prevalence, and years lived with disability for 301 acute and chronic diseases and injuries in 188 countries, 1990–2013: a systematic analysis for the Global Burden of Disease Study 2013. Lancet. 2015 8 22;386:743–800. 10.1016/S0140-6736(15)60692-4 Epub 2015 Jun 7. http://www.ncbi.nlm.nih.gov/pubmed/26063472. 26063472 PMC4561509

[3] Ribeiro I, Sevcsik AM, Alves F, Diap G, Don R, et al New, improved treatments for Chagas disease: from the R&D pipeline to the patients. PLoS Negl Trop Dis 2009; 3: e484 10.1371/journal.pntd.0000484 19582163 PMC2702098

[4] Garcia MN, Murray KO, Hotez PJ, Rossmann SN, Gorchakov R, et al Development of chagas cardiac manifestations among Texas blood donors. Am J Cardiol. 2015 1 1;115:113–7. 10.1016/j.amjcard.2014.09.050 Epub 2014 Oct 14. 25456877

[5] Garcia MN, Aguilar D, Gorchakov R, Rossmann SN, Montgomery SP, Rivera H, Woc-Colburn L, Hotez PJ, Murray KO. Evidence of autochthonous Chagas disease in southeastern Texas. Am J Trop Med Hyg. 2015 2;92:325–30. 10.4269/ajtmh.14-0238 Epub 2014 Nov 4. 25371187 PMC4347336

[6] Requena-Méndez A, Aldasoro E, de Lazzari E, Sicuri E, Brown M, et al Prevalence of Chagas disease in Latin-American migrants living in Europe: a systematic review and meta-analysis. PLoS Negl Trop Dis. 2015 2 13;9:e0003540 10.1371/journal.pntd.0003540 eCollection 2015 Feb. 25680190 PMC4332678

[7] Morillo CA, Marin-Neto JA, Avezum A, Sosa-Estani S, Rassi A Jr., et al Randomized trial of benznidazole for chronic Chagas’ cardiomyopathy. N Engl J Med 2015; 373(14):1295–306. 10.1056/NEJMoa1507574. 26323937

[8] de Andrade AL, Zicker F, de Oliveira RM, Almeida Silva S, Luquetti A, et al Randomised trial of efficacy of benznidazole in treatment of early Trypanosoma cruzi infection. Lancet. 1996 11 23;348:1407–13. 8937280 10.1016/s0140-6736(96)04128-1

[9] Sosa Estani S, Segura EL, Ruiz AM, Velazquez E, Porcel BM, Yampotis C. Efficacy of chemotherapy with benznidazole in children in the indeterminate phase of Chagas' disease. Am J Trop Med Hyg. 1998 10;59:526–9. 9790423 10.4269/ajtmh.1998.59.526

[10] Chippaux JP, Salas-Clavijo AN, Postigo JR, Schneider D, Santalla JA, Brutus L. Evaluation of compliance to congenital Chagas disease treatment: results of a randomised trial in Bolivia. Trans R Soc Trop Med Hyg. 2013 1;107:1–7. 10.1093/trstmh/trs004 23296694

[11] Molina I, Gomez i Pratt J, Salvador F, Trevino B, Sulleiro E, et al Randomized trial of posaconazole and benznidazole for chronic Chagas’ disease. N Engl J Med 2014; 370: 1899–908. 10.1056/NEJMoa1313122 24827034

[12] American Cancer Society. Cancer Facts & Figures 2015. http://www.cancer.org/research/cancerfactsstatistics/cancerfactsfigures2015/index. Accessed October 8, 2015.

[13] Fabbro DL, Danesi E, Olivera V, Codebo MO, Denner S, et al Trypanocide treatment of women infected with Trypanosoma cruzi and its effect on preventing congenital Chagas. PLoS Negl Trop Dis 8(11): e3312 10.1371/journal.pntd.0003312 25411847 PMC4239005

[14] Schijman AG, Bisio M, Orellana L, Sued M, Duffy T, et al International study to evaluate PCR methods for detection of Trypanosoma cruzi DNA in blood samples from Chagas disease patients. PLoS Negl Trop Dis. 2011 1 11;5:e931 10.1371/journal.pntd.0000931 http://www.ncbi.nlm.nih.gov/pubmed/21264349. 21264349 PMC3019106

[15] Drugs for Neglected Diseases Initiative. DNDi R&D Projects. http://www.dndi.org/diseases-projects/portfolio.html. Accessed October 7, 2015.

[16] Sabin Vaccine Institute. Chagas Disease/Leishmaniasis. http://www.sabin.org/programs/vaccine-development/chagas-diseaseleishmaniasis. Accessed October 7, 2015.

